# Rural and urban clinician views on COVID-19’s impact on substance use treatment for individuals on community supervision in Kentucky

**DOI:** 10.1186/s40352-024-00266-9

**Published:** 2024-03-26

**Authors:** Carrie B. Oser, Maria Rockett, Sebastian Otero, Evan Batty, Marisa Booty, Rachel Gressick, Michele Staton, Hannah K. Knudsen

**Affiliations:** 1 Department of Sociology, Center on Drug & Alcohol Research, Center for Health Equity Transformation, University of Kentucky, 1531 Patterson Office Tower, Lexington, KY 40506 USA; 2 Department of Sociology, Center on Drug & Alcohol Research, Center for Health Equity Transformation, University of Kentucky, 1515 Patterson Office Tower, Lexington, KY 40508 USA; 3 College of Medicine, University of Kentucky, 1515 Patterson Office Tower, Lexington, KY 40506 USA; 4 Department of Sociology, Center on Drug & Alcohol Research, University of Kentucky, 845 Angliana Avenue, Lexington, KY 40508 USA; 5 College of Public Health, University of Kentucky, 845 Angliana Avenue, Lexington, KY 40508 USA; 6 Department of Behavioral Science, Center on Drug & Alcohol Research, University of Kentucky, 117 Medical Behavioral Science Building, Lexington, KY 40508 USA; 7 Department of Behavioral Science, Center on Drug & Alcohol Research, University of Kentucky, 845 Angliana Avenue, Lexington, KY 40508 USA

**Keywords:** COVID-19, Clinician, Substance use disorder treatment, Rural, Urban, People on community supervision

## Abstract

**Background:**

The COVID-19 pandemic produced system-level changes within the criminal legal system and community-based substance use disorder (SUD) treatment system with impacts on recovery efforts. This study examines rural and urban clinicians’ perspectives of COVID-19 on SUD treatment delivery for people on community supervision.

**Methods:**

Virtual qualitative interviews were conducted between April and October 2020 with 25 community supervision clinicians employed by Kentucky’s Department of Corrections (DOC), who conduct assessments and facilitate community-based treatment linkages for individuals on probation or parole. Transcripts were analyzed in NVivo using directed content analysis methods.

**Results:**

Clinicians were predominantly white (92%) and female (88%) with an average of over 9 years working in the SUD treatment field and 4.6 years in their current job. Four COVID-19 themes were identified by both rural and urban clinicians including: (1) telehealth increases the modes of communication, but (2) also creates paperwork and technological challenges, (3) telehealth requires more effort for inter/intra-agency collaboration, and (4) it limits client information (e.g., no urine drug screens). Two additional rural-specific themes emerged related to COVID-19: (5) increasing telehealth options removes SUD treatment transportation barriers and (6) requires flexibility with programmatic requirements for rural clients.

**Conclusions:**

Findings indicate the need for community-based SUD treatment providers approved or contracted by DOC to support and train clients to access technology and improve information-sharing with community supervision officers. A positive lesson learned from COVID-19 transitions was a reduction in costly travel for rural clients, allowing for greater engagement and treatment adherence. Telehealth should continue to be included within the SUD continuum of care, especially to promote equitable services for individuals from rural areas.

**Supplementary Information:**

The online version contains supplementary material available at 10.1186/s40352-024-00266-9.

## Background

People with substance use disorders (SUD), including opioid use disorder (OUD), are vastly overrepresented in carceral and community supervision settings (CASA Columbia, 2010; Winkelman et al., 2018). Beginning in the 1990s, experts advocated for effective prison-based SUD treatment programs and post-release substance use aftercare initiatives (APA, 2004; Field, 1998; Martin et al., 1999; Wexler et al., 1999), which are associated with reductions in return to substance use, recidivism, health burdens, and economic costs (APA, 2004; Field, 1998; Martin et al., 1999; NASHP, 2019; Wexler et al., 1999). Despite the clear need for continuity of SUD treatment infrastructures for individuals on community supervision (i.e., probation or parole), adequate continuity of care has remained a challenge for many states (NASHP, 2019).

The need for expansive care networks for individuals on community supervision was further highlighted during the rapid spread of the SARS-CoV-2, the virus that causes COVID-19. The COVID-19 pandemic magnified substance use and opioid use adversities, as evidenced by increased rates of overdose deaths (Ahmad et al., 2019; Mumba et al., 2021). The COVID-19 pandemic exacerbated challenges in the delivery of SUD services in carceral settings due to space limitations and staff shortages (Nowotny et al., 2020). For individuals re-entering communities after release from carceral settings due to the pandemic as well as those already on community supervision, many experienced disruptions in medical and mental health care (LeMasters et al., 2023). Further, the pandemic was challenging for community-based SUD treatment providers, demanding a rapid transition from in-person services to virtual platforms to reduce COVID transmission risk and promote compliance with stay-at-home orders (Hirko et al., 2020; Mumba et al., 2021).

What is less clear is how the pandemic impacted the work of professionals at the intersection of the criminal-legal system and the community treatment system, particularly professionals working to coordinate services for those re-entering communities who have substance use-related health care needs. Such professionals have to navigate dual systems; they are employed by criminal-legal organizations, where they must collaborate with other staff such as probation and parole officers as well as help clients to navigate what is an often fragmented system of SUD treatment, requiring further collaboration. The ways that the pandemic may have impacted these types of inter- and cross-system collaborations have been under-described.

The rise of telehealth treatment options in community-based treatment because of the COVID-19 pandemic likely presents some advantages for individuals on community supervision, such as increased treatment accessibility, greater patient privacy, stigma reduction, and improved rapport between clients and clinicians due to the home-based environment of treatment (Kaur et al., 2022; Uscher-Pines et al., 2020). Since individuals on community supervision often experience barriers to healthcare due to discrimination (Redmond et al., 2020), participating in telehealth services could eliminate some of these barriers and support advantageous outcomes. However, challenges to telehealth have also been observed, including technology accessibility and chaotic home environments disrupting treatment (Hirko et al., 2020; Uscher-Pines et al., 2020).

The impacts of the COVID-19 pandemic on individuals under community supervision may differ between rural and urban areas. Telehealth may carry more relevant benefits for rural geographic locations (Edmunds et al., 2012; Hughes et al., 2021; Kaur et al., 2022; Kedia et al., 2021; Marcin et al., 2016). Many rural regions have limited treatment infrastructure and few healthcare specialists (Kedia et al., 2021; Marcin et al., 2016). In-person SUD treatment can also be costly in terms of money and time invested (Sigmon, 2014; Uscher-Pines et al., 2020). For example, Sigmon (2014) found that rural individuals allotted at least five hours a week and spent $50 on transportation to OUD services alone. For individuals on community supervision, lack of public transportation and driver’s licenses often inhibit the treatment process (Bunting et al., 2018), which suggests telehealth may improve treatment retention. However, rural areas continue to experience a digital divide with less access to reliable high-speed broadband networks (Lee et al., 2022). With these considerations in mind, understanding the pandemic’s impacts in both rural and urban areas is important.

The perspectives of clinicians are crucial in understanding how the COVID-19 pandemic impacted treatment services for individuals on community supervision. Studies indicate that clinicians working in OUD treatment perceive benefits to the efficacy and accessibility of telehealth (Riedel et al., 2021; Treitler et al., 2022) but also acknowledge concerns regarding the sustainability of such modalities, especially in terms of workload (Huskamp et al., 2022). In pre-COVID pandemic Appalachian Kentucky, clinicians serving individuals on community supervision already cited large caseloads as a barrier to effectively linking individuals to SUD treatment (Bunting et al., 2018), and telehealth might increase this burden. Clinicians also have first-hand experience in both in-person and telehealth modalities, allowing for them to offer comparisons in the perceived quality of SUD treatment. However, there are relatively few studies that have compared the perspectives of clinicians working in rural and urban areas, where the impacts of the pandemic may differ given pre-existing differences in the SUD treatment landscape as well as economic disparities experienced in many rural areas.

This study examines the perceptions of clinicians providing services to people on community supervision (i.e., on probation or on parole after release from prison) regarding treatment shifts and trends in the early months of the pandemic, particularly with regard to barriers and facilitators to care. Perceptions of clinicians working in rural and urban areas are compared to examine the ways in which these impacts may be similar or different, both for clients in needs of services and in how the pandemic affected clinicians’ work experiences. Although the pandemic has evolved since its initial impacts in 2020 and public health threats have lessened, consideration of these impacts is still warranted as they may inform ongoing nationwide efforts to address health inequities experienced by people on community supervision.

## Methods

### Participants

As part of an NIH-supported study titled Geographic variation in Addiction Treatment Experiences (GATE), in-depth qualitative interviews were conducted with social service clinicians working within Kentucky’s Department of Corrections (DOC). Additional details about the GATE study protocol are outlined elsewhere (Oser et al., 2023). The Kentucky DOC oversees all adult carceral institutions (i.e., Division of Adult Institutions) and all adult community supervision offices (i.e., Division of Probation and Parole) within the state. In addition, the Kentucky DOC’s Division of Addiction Services provides clinical and administrative oversight for the provision of SUD treatment in carceral institutions as well as the screening, assessment, medication-assisted treatment (i.e., extended-release naltrexone, buprenorphine) and linkage to community-based SUD treatment for individuals on community supervision.

Social service clinicians, herein referred to as clinicians, are employed by the Division of Addiction Services and are typically located in community supervision offices to provide an array of non-clinical services to people on probation or parole. Clinicians meet with clients, who can be referred by a variety of DOC staff or via self-request, to conduct screening, assessments and periodic check-ins (e.g., monthly) and to link clients to DOC-approved, contracted, and additional community SUD treatment providers and recovery support groups across the state. Clinicians can recommend a change in the treatment plan based on the clients’ assessments, clinical needs, or personal requests (e.g., a positive urine drug screen may warrant an assessment, and subsequent recommendation for a higher level of care according to the American Society for Addiction Medicine [ASAM] criteria). Ultimately, clinicians are tasked with promoting continuity of care for clients who completed either community SUD treatment or prison-based SUD treatment (prioritizing the increasing number of clients who initiated medication for opioid use disorder (MOUD) while in prison) and providing additional case management services.

### Procedures

The DOC’s Division of Addiction Services provided a list of contact information for all clinicians working within Kentucky’s 54 Appalachian counties, as defined by Appalachian Regional Commission (2021), and the two largest urban counties (Jefferson county, which includes the city of Louisville; Fayette county, city of Lexington). Using a census sampling approach, all 29 clinicians on this list were invited to participate in a virtual (i.e., Zoom) or phone-based interview and 25 enrolled in the study between April and October 2020, resulting in an 86% participation rate. Of the participants, 18 (72%) clinicians were employed in a rural Appalachian county.

Before data collection, trained GATE staff obtained informed consent using a waiver of documented informed consent process. The interviews lasted about one hour and were audio-recorded. Participants were offered a token of appreciation (valued at less than $10) for their participation as monetary incentives for staff are prohibited by the Kentucky Executive Branch Ethics Commission. All procedures were approved by the University of Kentucky’s Institutional Review Board and participants were protected by a Certificate of Confidentiality. No individual-level data was shared with the DOC.

### Qualitative interview guide

The GATE study, including the qualitative interview guide, was developed using the social ecological framework (Brofenbrenner, 1979; McLeroy et al., 1988), with a focus on rural-urban differences. The interview guide (see Appendix A) included questions about the factors (i.e., individual, interpersonal networks, and structural) that affect client treatment outcomes, especially as they related to clients who initiated MOUD in prison and transition back to both rural and urban communities. COVID-19 emerged as a public health crisis in 2020, prior to fielding the qualitative data collection, so two additional questions were included to examine the impact of the pandemic from the clinicians’ perspectives on their jobs and clients’ substance use and recoveries.

### Analytic plan

Immediately following the interviews, GATE staff transcribed audio recordings. Individuals, including participants, were not identified on the transcripts. The project director led a coding team comprised of two coders trained in qualitative research methods. Using NVivo 12.0 software, each coder applied initial primary codes aligning with the overarching social ecological model to the same transcript. Next, coders used an inductive process to add preliminary secondary codes to create an initial codebook. The team then applied the initial codebook to the same transcripts to identify additional secondary codes emergent in the data. Three transcripts were coded until no new secondary codes were found, resulting in the final codebook. Then each coder recoded those initial three transcripts using the finalized codebook and the recoded transcripts were compared for inter-coder consistency. Discrepancies were resolved through discussion during a qualitative team coding meeting, but when consensus could not be reached, the project director made the final decision. This process continued for the coding of the remaining transcripts. Double-coding was used to assess the intersections between codes across the individual, interpersonal networks, and structural factors affecting client treatment outcomes. For this study, we examined the primary code of “COVID,” including the secondary codes of “program compliance” and “virtual workplace,” with specific attention on the how these codes intersected with the primary code for the clinician’s “geographic location” (i.e., rural or urban service area).

## Results

Table 1 shows descriptive statistics on the socio-demographic backgrounds of the clinicians who participated in the study. Four themes related to COVID-19 were identified from the qualitative interviews relevant for rural and urban clinicians. These themes included the transition to telehealth increases modes of communication, creates paperwork and technological challenges, requires more effort to collaborate, and creates concern about having less information about their clients. In addition, the qualitative analyses identified two COVID-19 themes relevant to rural clinicians only, including COVID-19’s positive effect on rural clients’ virtual attendance at SUD clinical appointments due to the removal of transportation barriers and clinician flexibility with program requirements for rural clients. No urban-specific themes were identified. Illustrative quotes were selected from the transcripts to support each theme, which are framed as lessons learned below.

**Table 1:** Descriptive statistics of clinicians working with clients on community supervision (*n* = 25)

		%	Mean (Standard deviation)	Range
Rural		72%		
Female		88%		
White		92%		
Age			40.6 (9.56)	29–70
In recovery		8%		
Have family with SUD		80%		
Education				
	Master’s degree	48%		
	Bachelor’s degree only	52%		
Type of clinician				
	Certified clinician	52%		
	Licensed clinician	12%		
Career experience				
	Number of years in SUD treatment		9.3 (8.3)	2 months to 30 years
	Number of years in current position		4.6 (7.6)	2 months to 30 years
Number of clients			124.4 (59.4)	15–252

### Telehealth increases modes of communication with clients

The move away from in-person interactions was mentioned by almost all of the clinicians (*n* = 21) and resulted in benefits for clinicians as well as their clients. Clinicians mentioned the initial challenge of learning new technology but also highlighted the benefit of this new skillset. They had limited time to prepare for the transition from working in the community supervision offices to working from home but understood the need for this safety precaution. While the virtual communication platforms, such as Zoom, had a learning curve for clinicians, they were able to quickly adapt and expressed gratitude for the opportunity to learn new skills. A rural clinician said, *“We were just told, ya know, that we were going to be working from home and I wasn’t sure how that was gonna work out, especially with scanning documents and things like that, but actually, it’s been great. I’ve learned a lot about technology that’s for sure. And I really, I kinda like it. It’s a more relaxed atmosphere and conversation just seems to flow a little easier.”*

The DOC provided clinicians with state cell phones, which benefited them and their clients. Many clinicians served multiple counties and thus, spent a great deal of time traveling to their offices in different counties pre-COVID. For clinicians who may be new in their role, the absence of state cell phones posed a challenge for clients to reach the clinician if they were not in their office and text messaging was not an option. This point is articulated by one recently hired rural clinician, “*I think that I’ve been able to connect more with them [clients], because they have different access to me, as far as now I have a state phone. […] Now they all have access to me, and there’s a different element to the fact that they can text me on the state phone, so they kinda like that as well. Because they don’t have to come in and physically talk.”* While telephonic communication was the primary way that clinicians contacted their clients during COVID-19, the pandemic broadened the number of modes that clinicians used to connect with their clients in both rural and urban areas which was beneficial. Both text-messaging and virtual communication were used more frequently.

### Telehealth creates paperwork and technological challenges

The transition to work from home created some challenges for clinicians, including increasing the time that it took to complete routine job duties and requiring clinicians to help their clients navigate difficulties with technology. For example, virtual work entailed scanning more documents and developing technological solutions to virtually obtain signatures on required forms. The additional time to obtain required signatures or complete paperwork was a source of aggravation. A rural clinician describes this by saying “*[T]ypically we have signature pads and electronic names of the clients sitting across the desk from me where we would sign paperwork. […] Lots of them don’t have email or don’t know how to set-up an email. So, I’m spending a great deal of time on the phone explaining forms and things like that.”* In addition, creating online resources for clients was discussed, and a rural clinician provided an overview of this process during their interview. They said “*We made online recovery resources packet and filled it up as much as we could. I have been personally recommending intherooms.com, it’s pretty user-friendly and I went through and made a big picture step-by-step tutorial about how to use it and get logged on*.” Clients could use these online recovery resource packets at any time; however, they did require an additional investment of time for clinicians to generate during the stressful transition to virtual work.

During stay-at-home orders at the beginning of the pandemic, clinicians tried to assist their clients with addressing technological limitations and accessing needed resources (e.g., phone, internet) to promote their recovery journeys. Many clients, especially those who were older or had less formal education, did not have basic computer literacy skills, which made virtual communication, outside of phone calls, extremely complicated. Clinicians shared the challenge of clients running out of minutes on their cell phone plans or not having consistent internet access, which limited their clients’ ability to fully engage in treatment sessions and recovery support services. A rural clinician pointed out that “*We had to help all these, the folks that are in classes get email addresses, which a lot of them didn’t have. Help them get the Zoom app on their phone which some of ‘em, it was difficult for some of them, so we had to get everybody set up to use telehealth which was a big hurdle*.” A clinician working in a rural county denoted the need to be resourceful in addressing challenges with internet access:

*[H]e lives out in the country, like no service, nothin’, and so we finally just had to have a conversation with him like, “Look, your attendance is imperative, you have to be there, you have to sign on if you get disconnected, you have to log back in, so we finally just told him yesterday if you have to go sit in your vehicle in a McDonald’s parking lot, that’s what you have to do.”*

Reaching clients via phone was also more difficult during COVID’s initial impact in 2020, and clinicians reported having to make numerous attempts to connect with clients. In fact, some clinicians made additional efforts to check-in with clients more frequently if they knew clients had limited internet access. As illustrated in the quotes above, clinicians found these hurdles to be a challenge, but surmountable and short-term solutions were identified. Again, the transition to virtual communication created additional challenges for clinicians and their clients alike.

### Telehealth requires more effort to collaborate

The onset of COVID-19 in 2020 and the resulting rapid organizational changes within the DOC, including among community supervision branches, highlighted the need for collaboration both within their agency and with community SUD treatment centers to address virtual communication barriers. This theme was embraced among both urban and rural clinicians. While all clinicians were employed within the Kentucky DOC’s Division of Addiction Services, it was clear that the different divisions within the DOC quickly came together to promote the transition to virtual work and ensure clients on community supervision had access to needed services. A clinician from a rural county explained their perspective in saying “*We have the best support system as far as our team. Like, our division, we all work together. I’ve never worked in a place where, if somebody is handed some type of resource it’s distributed over the entire state to all of us.”* Clinicians noted that virtual communication barriers were addressed by DOC’s provision of state cell phones and leveraging of virtual communication platforms, such as Zoom. In addition, they noted the legal department within DOC allowed clinicians to review documents with clients via phone.

Regarding collaboration between clinicians and community supervision staff, it was noted that working in the community supervision office together resulted in more in-depth, meaningful discussions about clients’ needs and more rapid responses than working from home. For example, in explaining their partnership pre-COVID, a rural clinician said, “*So, if I suspected something I could just get an officer to, you know, perform a drug screen on the individual*.” Because community supervision officers interacted with clients more regularly, clinicians relied on officer insights on clients, which was now occurring by phone or email. Clinicians emphasized that cross-division collaboration still occurred, but the pandemic slowed the pace.

In addition, clinicians across the state explained the need for greater collaboration with SUD treatment centers during COVID, which offer a range of services (e.g., intensive outpatient care, MOUD treatment). At the onset of COVID-19 in 2020, SUD treatment centers had to rapidly change how they delivered care, like other healthcare agencies. Clinicians spoke of SUD treatment center closures and limited residential and intensive outpatient program (IOP) treatment slots, highlighting their struggles to find clients an appropriate level of care based on their assessment. When the pandemic restricted access to higher levels of care, clinicians sought additional treatment options. An urban clinician explained that it was critical to get people engaged in any level of care during this unprecedented global health crisis: *“[M]aybe they weren’t at the point that they needed that residential piece yet, but they definitely needed IOP that we couldn’t provide that to ‘em, we at least got them in to individual counseling.”* Clinicians were working hard to connect clients to needed SUD services and were eager to collaborate with SUD treatment centers, including DOC-contracted, approved, and other community providers, to ensure client needs were met.

However, clinicians recognized that some clients needed more intensive care and were unlikely to receive it in a timely way. A clinician from an urban county shared the difficulties they experienced by stating “*And it is hard, when treatment facilities are not accepting that many people, or they’re shutting down completely. So my options for them are pretty limited right now.”* Rural clinicians also had these experiences with communication barriers and highlighted detrimental outcomes that resulted from waiting lists, limited collaboration with SUD treatment centers, and limited communication with clients’ families due to inaccessibility and HIPAA confidentiality protections:

*I had two overdoses, deaths, on my caseload …. And, so, one of them had left treatment. Treatment never told us what happened and they died a week later, and I didn’t know that they had even like treatment. So that was like a miscommunication on the treatment part of the facility. And then I think another one had passed away from an overdose and it’s one of those things where, like normally if a family member notices a change they’ll let me know.*

Clinicians, regardless of geographic location, perceived collaboration with the SUD treatment providers as key to ensuring their clients’ treatment attendance and engagement. If a client was missing treatment sessions, SUD treatment providers needed to notify the clinician working in the community supervision office so that multiple entities reached out to the client to let them know they care about their recovery.

Clinicians from both rural and urban areas discussed the impacts of the state’s rapid release of people from prisons and jails who were incarcerated for non-violent class D and misdemeanor offenses to prevent the spread of COVID-19, a phenomenon happening nationwide. Many of these individuals had a SUD and needed treatment as they were transitioning from jail back to their home communities. An urban clinician explained their collaboration with jails, community supervision, and SUD treatment centers to address the rapidly expanding population of newly released individuals:

*A lot of people were in county jails waiting on beds and those were kinda my main focus, like, “Let’s get them out of jail and then we’ll work on the community.” [I]nstead of just saying, “Hey, sorry, you’re gonna have to figure it out for yourself,” we were able to request that, like places in our community do that individual telehealth and just kinda let that count toward sessions, or at least keeps these people engaged somehow, in some form of treatment.*

While clinicians regularly interacted with treatment centers to arrange client placements as part of their position, this need was heightened during the pandemic due to the limited treatment slots, SUD treatment center closures, and/or health concerns about in-person SUD treatment. A rural clinician noted “*lot of our facilities that we have DOC contracts with, they’ll take individuals straight out of the jails, because they know they’ve been kind of quarantined in a sense, versus individuals straight from the community because of that threat of them possibly spreading coronavirus*.” SUD treatment centers’ preferential selection of clients from jails over those on community supervision was a concern of this clinician.

### Telehealth limits client information

A widespread need experienced by clinicians during the COVID-19 pandemic was improved communication with clients to increase the amount of information available to inform their clinical decision-making and further strengthen their ability to establish rapport. Both rural and urban clinicians emphasized that it was difficult to conduct a thorough assessment over the phone. Before the pandemic, many experienced clinicians reported the assessment of non-verbal, visual cues to inform their clinical decision-making. However, it was noted that during the pandemic, clinicians could not gauge visual cues of client wellness via phone sessions and instead solely relied on verbal client self-reports. An urban clinician outlined how their assessment process changed during the COVID-19 pandemic and the lack of face-to-face contact limited available information: *“And I feel like that face-to-face contact, and eye contact, and observation of mannerisms really does lend for establishing a better rapport.”* Rural clinicians echoed this sentiment. It should be noted that clinical decision-making should not solely rely on non-verbal visual cues alone, and the role of stigma and bias in assumptions of client substance use should be critically considered. A myriad of factors should be assessed to provide quality clinical care (e.g., evidenced-based skills, interpretation and use of diagnostic tests such as urine drug screens, an understanding of cognitive biases, etc.).

There was agreement that it was easier to establish rapport during face-to-face meetings with clients. An urban clinician conveyed:

*If they were sitting across from me in my office I could say, “you say you’re doin’ well, but you’re not smiling, or you don’t seem confident when you say that, or your body language says somethin’ else.” And I can usually get them to open up to me. I think once they realize that I’m not an officer, I can’t arrest them, they are more willing to open up and talk to me… And usually by the time they’re leavin’ my office it’s, “I’m sorry to unload on ya.” And it’s not unload, like, I really enjoy being able to give them resources that can continue to help them with mental health and substance abuse.*

Because clinicians worked in community supervision offices, there was often an incorrect assumption that they were “trying to catch” the client doing something wrong. However, the clinicians’ job duties were focused on ensuring that clients had needed resources to support their recoveries. If clients returned to substance use, the clinician could reassess and attempt to link them to the appropriate level of care for their SUD.

COVID-19 also resulted in the reduced frequency of community supervision staff conducting random and/or periodic urine drug screens for clients. Urine drug screens were still available if there was officer concern, clinician concern, or self-reported drug use. Clinicians viewed drug screen results as a useful tool in evaluating if clients were accurately reporting substance use or in need of a higher level of care for their SUDs. A rural clinician shared “*It’s a lot harder, especially with, seeing if people are actually using or not. A lot of times we base that on drug screens and things like that and we can’t really do that right now. So of course when you call people, “No I haven’t been using,” that’s the typical answer you get. And a lot of times that’s probably true, but we know that it’s not always accurate*.” Urban clinicians also expressed concern with clients’ return to use during the pandemic, with one urban clinician saying “*And I know, whenever that time come when we start back, it’s gonna be a lot of [positive urine drug screens], I’m sure.*”

Despite the challenges caused by having less visual and objective information on their clients during COVID-19, it was clear the clinicians were devoted to ensuring their clients were set up for success. Many clinicians were concerned about making appropriate treatment recommendations without non-verbal cues that could be gathered during in-person interactions and urine drug screen results. Pre-COVID, the in-person interactions fostered rapport, and along with urine drug screens, provided an additional layer of accountability. Moreover, this seemed especially important for clients who were recently released from the hyper-structured environment of prison. To address this challenge, clinicians underscored the need to engage in active listening and that they had “*to re-learn how to hear what people were saying without seeing them*,” as shared by a rural clinician.

### Increasing telehealth options removes SUD treatment transportation barriers for rural clients

Only rural clinicians discussed the positive impact of COVID-19 on their clients’ appointment attendance due to increased telehealth options, resulting in this geographically-specific theme that emerged from the qualitative interviews with the 18 rural clinicians. Over half of rural clinicians noted that transportation was often a barrier to SUD treatment, especially in IOP treatment that required rural clients to travel long distances at least three times per week for multi-hour treatment sessions. Due to COVID-19, many SUD treatment centers transitioned from in-person office-based treatment to telehealth options that allowed more clients to safely participate in services from the comfort of their homes. This was a silver lining of the pandemic as a rural clinician said, “*If we take that transportation barrier down, a lot of our clients are being more successful for at least attending the sessions*.”

Rural people on community supervision faced many transportation challenges, which represented a barrier to complying with treatment program and supervision requirements. Many lacked a valid driver’s license or did not have access to an insured vehicle, resulting in individuals relying on family or friends to travel to their SUD treatment appointments. Ride-shares and public transportation were often nonexistent in rural counties; and if ride-shares such as Uber or Lyft were available, they were cost-prohibitive. A rural clinician further highlighted how expensive transportation could be by sharing, “*It’s a constant problem here, because we have some individuals that will pay fifty dollars for a 25-minute ride. And that’s an everyday thing.*” In this example, if the client was in an IOP that required in-person session attendance three times per week, the client would be paying an extra $300/week for transportation to SUD treatment. Telehealth helped this client enter and remain engaged in treatment. Another rural clinician expressed their view:

*Before you had to always come to the office and if you don’t have a car, you don’t have a driver’s license, and you’ve burned every bridge in your family, that’s just almost impossible where we live. So, from what I’ve been told is that from now on out they’re gonna be more willing to work with people with telehealth and I think that is absolutely wonderful and it surprises me that it took a pandemic to get there.*

A lesson learned was that virtual attendance and participation in SUD treatment was deemed beneficial during the pandemic. As illustrated in the quote above, rural clinicians desired the continuation of telehealth options to promote equity in engaging rural clients in SUD services.

### Flexibility is needed with programmatic requirements for rural clients

Another rural-specific theme that emerged from the qualitative data with the 18 rural clinicians was the need for creativity or leniency with programmatic elements for rural clients on community supervision during the pandemic. Rural clinicians noted numerous challenges often faced by individuals as they transition from prison to the community (e.g., obtaining state identification, re-enrolling in health insurance, and/or securing employment) and these challenges were exacerbated during COVID-19. Shelter-in-place orders and government office closures limited access to needed documents (i.e., state ID cards or driver’s license), which further negatively affected the opportunity to gain employment. Moreover, industries more likely to hire people with criminal records, such as retail, entertainment, and food service industries, were closed or provided limited services for a period. Further, these industries were public-facing and thus increased people’s exposure to COVID-19.

Clinicians were empathetic with rural clients, especially those with limited economic resources and increased economic stressors, and they tried to identify creative ways to support clients in their recovery journeys. Examples included attendance at virtual mutual support groups, accessing online educational resources, and journaling to meet aftercare or treatment programmatic requirements. One rural clinician shared:

*Well, it’s, it’s allowed for some creativity […] I call just breaking it up into different categories to, you know; “What are you doing for your recovery? What are you doing to show that you’re being responsible?” And you know, one of those things is going to get your driver’s license, or going to get your ID, or going to get your insurance and you know, you can’t do those things. And for many of our individuals, they are not able to become employed. But there’s a lot of those things the participant can’t do in order to earn points so it’s caused me to have to be creative and it’s caused the clients to have to kinda get creative. What I’ve been doing is using journaling and logging of emotions and reading literature on the internet[.]*

During the pandemic, rural clinicians emphasized the lesson they learned in finding new ways of navigating DOC and SUD treatment centers procedures to “meet the client where they are.”

## Discussion

This study identified six themes regarding clinicians’ perspectives of the impacts of the COVID-19 pandemic on people on community supervision who have SUD. The themes, especially the two rural-specific themes, shed light on lessons learned by clinicians to create more equitable care in rural areas. Moreover, although the interviews asked broadly about impacts of the pandemic, clinicians centered telehealth in their descriptions of how their work and how the treatment environment evolved during the early phase of the COVID-19 pandemic (i.e., April to October 2020). Even though many of the most deleterious health impacts of the COVID-19 pandemic have waned since 2020 with the availability of vaccines and improved treatments, the findings about clinicians’ perspectives regarding telehealth have ongoing relevance for two reasons. First, many of the federal and state policy changes that allowed for SUD and OUD treatment via telehealth have been extended and remain in place (Agniel et al., 2023; Substance Abuse and Mental Health Services Administration, 2023). Second, telehealth has the potential to support the development of a more equitable system of care for individuals on community supervision, who face multiple health-related and economic-related disparities.

Rural clinicians noted how telehealth seemed to improve treatment attendance among their clients on community supervision. Those living in rural communities often face exacerbated rates of various chronic health conditions alongside treatment barriers such as lack of transportation, provider shortages, and lack of economic resources (Hirko et al., 2020; Kedia et al., 2021; Marcin et al., 2016). Specifically, individuals on community supervision in rural areas, who are more likely to have SUDs, may face even more substantial barriers to care (Zaller et al., 2022). Implementing telehealth within regions facing these barriers during COVID-19 seemingly widened treatment accessibility and utilization, which is consistent with other studies that have considered how telehealth in rural communities can address healthcare inequities and improve SUD treatment retention (Edmunds et al., 2012; Hughes et al., 2021; Kaur et al., 2022; Sistad et al., 2023). Of note, qualitative findings from Zaller and colleagues (2022) indicate that individuals on community supervision found telehealth options helpful, and they reported less treatment anxiety while receiving care on virtual platforms.

Although attendance benefits were reported by rural clinicians, there were concerns regarding the quality of care provided through virtual platforms, due to a lack of client information such as body language and visual cues. Shachak and Alkureishi (2020) acknowledge the lack of acquired client information on some virtual platforms and suggest the importance of moving from telephonic to video-based platforms. While studies indicate that telehealth services for substance use are as effective as in-person modalities (Batastini et al., 2016; Gliske et al., 2022), further research is needed to improve telehealth processes to better capture client information and to ensure equitable treatment outcomes for individuals on community supervision (Hirko et al., 2020; Schachar et al., 2020).

In order to maintain the accessibility benefits of telehealth while mitigating potential reductions in quality of care, improvements to telehealth infrastructure and training should be considered. Clinicians in this study report some technological barriers to completing telehealth services including lack of internet access, low technology competency, and financial burdens (e.g., running out of paid cell-phone minutes). In order to increase the success and sustainability of telehealth services, particularly in rural communities, it is essential that telehealth infrastructure is funded and supported to relieve this burden for clients (Vigilone & Nguyen, 2022). There is also a need to close the rural broadband divide, with a federal governmental infrastructure investment (Lee et al., 2022). These findings, among themes in this study, indicate that investing in high-speed internet infrastructure, appropriate technology, and training for underserved areas could bolster the reported benefits of telehealth services.

Another avenue for further exploration is the use of hybrid treatment modalities in order to increase attendance and accessibility while still providing opportunities for in-person meetings and the acquisition of physical observations. Kedia and colleagues (2021) suggest a hybrid care model in relation to serving rural Appalachian communities in order to mitigate geographical barriers to care. Of note, Kentucky DOC’s recent effort to address the geographic and transportation challenges faced by clients on community supervision includes a new ride assistance pilot program with the Kentucky Transportation Cabinet in which clients can request transportation to certain approved appointments, treatments, and classes (Tillson et al., 2023). Clinician reports from this study indicate a potential trade-off regarding telehealth appointment attendance and lack of client information, suggesting a need for improved technologies and the exploration of mixed modalities of treatment delivery.

In addition to technological barriers and the inability to visually assess clients, clinicians report increased difficulty in treatment collaboration efforts with other clinicians via virtual platforms. Namely, clinicians cite that the accessibility and quality of collaboration efforts are negatively impacted due to a lack of in-person meetings. Tuckson et al. (2017) note that telehealth includes not only services to connect clinicians and clients, but also provides a platform for clinician collaborations. For clinicians serving people on community supervision, additional teleservices connecting clinicians to probation and parole officers could prove beneficial for clients. Addressing technological and training barriers to virtual collaboration efforts could assist in more efficacious outcomes, although more research is needed in this area.

In further considering the impacts of virtual treatment modalities, it is also crucial to assess the implications for clinicians in terms of how these modalities impact their workload. The concerns around workload intensity align with other research. For example, Shachak and Alkureishi (2020) note that telehealth may intensify clinician burnout through screen fatigue, loss of information due to treatment modality, and due to challenges addressing difficult situations virtually. Telehealth often results in greater accessibility, which functionally increases clinician workloads (Kedia et al., 2021). Workloads also increase the extent to which clinicians need their clients to learn to use telehealth technology. However, the reduced burden on travel time and the ability to work remotely from home alleviates some of this burden. More research is needed to understand how telehealth impacts clinician well-being and burnout.

Several limitations should be noted. One limitation of this study is the small sample size of 25 clinicians and the population being limited to clinicians who work within one state’s community supervision offices. However, it is worth noting that having a clinician co-located in a community supervision office to focus on assessment and SUD treatment linkages is novel and is a model worthy of consideration for other states. Lessons learned from these clinicians may be beneficial to other states community supervision programs to improve SUD treatment to people on community supervision, especially in rural areas. It may be valuable to expand the study population to all SUD treatment clinicians in Kentucky to increase sample size and determine if the experiences described in this study are specific to treatment in criminal legal system settings. It should also be considered that the sample had a significant variance in caseloads ranging from 15 to 252 clients per clinician. However, it should be noted that the smaller caseload may reflect new staff or specialized caseloads, while the larger caseloads may reflect a clinician covering an additional district temporarily. Future research should examine the differences in clinician experiences with telehealth SUD treatment based on caseload to determine if variance influences telehealth treatment attitudes and efficacy.

Although the rapid transition to telehealth-focused treatment created some barriers to care, telehealth capabilities generally increased access, especially in rural areas. Some of the adjustments made in SUD treatment due to the pandemic can be used going forward to improve treatment access, especially if technology becomes more widely available in rural areas and more user-friendly. By assessing the impact of changes in SUD treatment based on clinician experiences, long-term changes can be made to improve treatment systems with specific tailoring to geographic location to promote health equity.

### Electronic supplementary material

Below is the link to the electronic supplementary material.

Supplementary Material 1

## Data Availability

The data generated from and analyzed in this study are available from the corresponding author on reasonable request.

## Abbreviations

SUD: Substance use disorder
OUD: Opioid use disorder
DOC: Department of corrections
COVID-19: Coronavirus caused by the SARS-CoV-2 virus
P&P: Probation and Parole
GATE: Geographic variation in addiction treatment experiences
MOUD: Medication for opioid use disorder
IOP: Intensive outpatient program
