# Supplementary material for: Rural and urban clinician views on COVID-19’s impact on substance use treatment for individuals on community supervision in Kentucky
Source: Health Justice. 2024 Mar 26;12:12. doi: 10.1186/s40352-024-00266-9 (PMC10964696; doi:10.1186/s40352-024-00266-9)
Supplement: Supplementary file 1 — Supplementary Material 1 [file 40352_2024_266_MOESM1_ESM.doc]

**Appendix A**

**
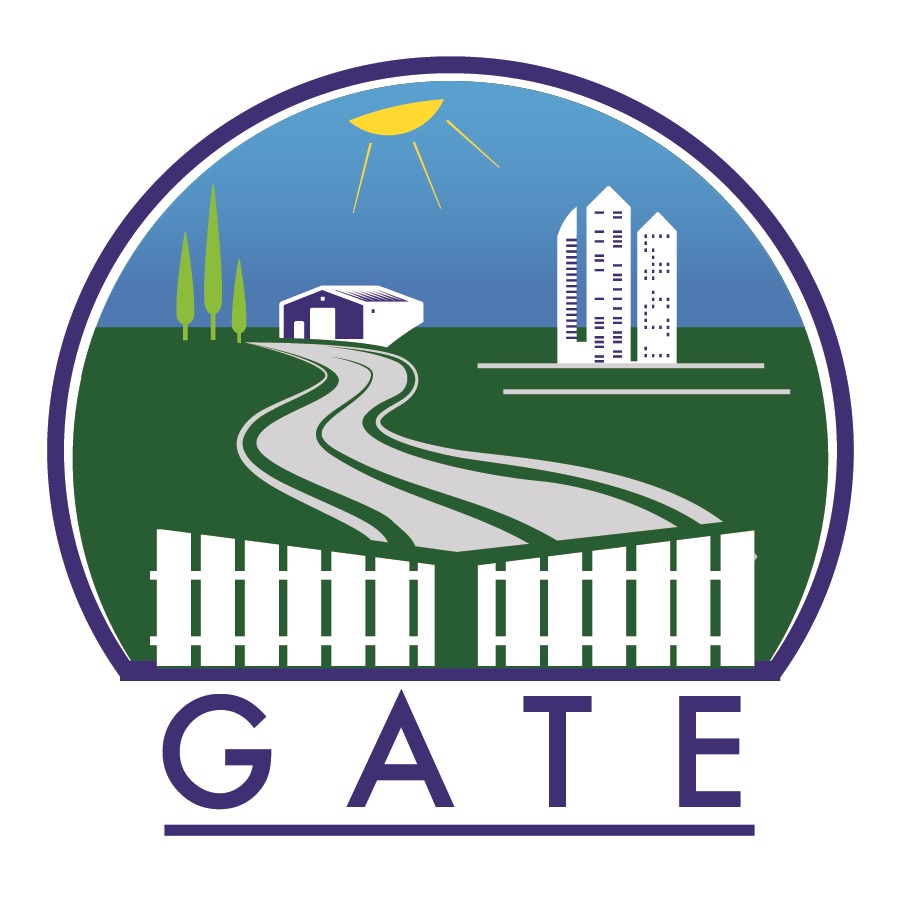
**

**Qualitative Interview Script:**

**Social Service Clinicians**

**At the start of recording the interview – please read:**

This interview is being conducted for the GATE Study. I am interviewing study participant number [PTID] on [date of interview] at [time of interview]. Before we begin please acknowledge that you give permission to audio record this interview by saying 'I agree to audio recording.'

Wait for Social Service Clinicians (SSC) to respond and read below.

**INTRODUCTION**

***Interviewer read:*** *Thank you for joining me today. I am with the Center on Drug & Alcohol Research at the University of Kentucky.*

***Purpose:*** *This qualitative interview is part of a new and exciting research study which is funded by the National Institute on Drug Abuse (NIDA). The purpose of this interview is to gain diverse perspectives on views of the three FDA approved medications for the treatment of opioid use disorder, including extended release injectable naltrexone (also known as Vivitrol), buprenorphine (Suboxone, Zubsolv, Sublocade), and methadone. Since Vivitrol and buprenorphine are offered as a treatment option in certain prisons, we will be asking questions to get information on the ways in which SAP clients are educated about Vivitrol and buprenorphine, the different factors that influence the initiation of these medications for opioid use disorder, and whether these factors vary geographically. Ultimately, we want to identify potential strategies to improve the prison delivery of these two medications and continued use after release by individuals re-entering both rural and urban areas. We want your opinions about the factors that impact client treatment outcomes from your perspective as a social service clinician, especially as they relate to clients who initiate Vivitrol or buprenorphine while incarcerated and then transition back to the community. Your opinions and insights will help inform future process improvement efforts for the continuum of care for clients with opioid use disorder. Thank you in advance for your participation.*

***Agenda:*** *This is a one-time interview that will last about one hour.*

***Confidentiality:*** *You are not required to answer any of the questions, and your answers will be kept confidential. This discussion is being audio-recorded so that we can have a complete record of what you have to say. Keep in mind, however, that all comments are confidential and used for research purposes only. Please do not use your name or the name of any other colleagues during this interview. It’s okay to say someone’s job title (e.g., Supervisor) instead of their name. No answers will be associated with your name. Your comments will be combined with all other responses so you will remain unidentified in any publications or reports to the KY Department of Corrections (DOC). Therefore, it is very important for you to be as honest as possible during this interview.*

**QUESTIONS**

1. We’d like to begin by asking you to describe your career path and how you became an SSC.

*Possible prompts:*

- - - *Please tell me more about your career path.*
    - *What kinds of jobs did you have before becoming an SSC?*
    - *What interested you about becoming an SSC?*

1. We’re currently experiencing a public health pandemic due to the novel coronavirus (COVID-19). Please describe if and how it has impacted your workplace, including your ability to do your job.
2. In what ways, if any, has COVID-19 affected your clients’ substance use and recovery efforts?
3. Now we want to focus on your interactions with clients with opioid use disorder. How does working with clients with opioid use disorder (OUD) differ from working with clients with other primary substance use order diagnoses? In what ways is it similar to working with clients with other primary substance use disorder diagnoses?

***Interviewer Read****: Here is a handout that describes each of the three types of medications for OUD. (Give participant Handout A).*

VIVITROL SECTION

1. We would like to discuss the three types of medications for the treatment of opioid use disorder. First, we would like to specifically discuss **Vivitrol**, and then we’ll move on to discuss buprenorphine and methadone. As part of the state’s response to the opioid epidemic, Senate Bill 192 was passed in 2015 to earmark funds for the DOC to offer Vivitrol to eligible people incarcerated prior to release from prison.

1. Describe your experience and/or training with Vivitrol prior to the passing of Senate Bill 192 in 2015.
2. What are your views on Vivitrol as a treatment for opioid use disorder?
3. In what ways does Vivitrol help the recovery process for clients with OUD?
4. In what ways does Vivitrol hinder the recovery process for clients with OUD?
5. Will you please tell me about any educational materials or training you received on Vivitrol? These could be trainings from DOC on the SAMAT protocol or pharmaceutical companies (e.g., Alkermes).
6. What additional educational materials, trainings, or resources, if any, do you need related to Vivitrol?
7. We’d like to go through a series of questions related to your experience with SAP clients transitioning from prison to the community. These questions are related to different individual factors that influence initiation and/or continued use of Vivitrol. By individual factors, we mean barriers or facilitators within the clients themselves, or client characteristics.
   1. For those clients who initiate Vivitrol in prison, what are some of the barriers within clients themselves that prevent them from continuing Vivitrol in the community?
   2. For clients who do continue Vivitrol in the community, what client characteristics help them to continue to receive this treatment?
   3. What types of things do clients say about side effects or other health concerns related to Vivitrol use?
8. These next questions are related to SAP client’s social networks. By client’s social network, we are referring to the people your client’s live, work, or socialize with on a regular basis.
   1. What role does the client’s social network play in preventing clients from continuing Vivitrol in the community?
   2. What role does the client’s social network play in helping clients continue Vivitrol in the community?
   3. How educated are your client’s family and friends about Vivitrol?

*Possible prompt:*

- - - *What information do people in your client’s social networks have about the costs, side effects, and potential risks and benefits of Vivitrol?*
  1. How common is it for your clients to have family members and friends who also have OUDs?
  2. What are the challenges your clients experience in creating and maintaining supportive relationships?

*Possible prompt:*

- - - *Are there mutual support groups (e.g., AA and NA meetings) in your clients’ communities?*

1. The next set of questions is focused on structural and institutional level factors that may affect the initiation and continued use of Vivitrol for clients transitioning from prison to the community. By structural and institutional factors, we mean things that are larger than the individual or families - like policies and procedures within the KY Department of Corrections, the legal system, healthcare organizations, or insurance companies. We also mean things like services and resources provided by local government and educational institutions.
   1. What are the barriers within the health care system to clients continuing Vivitrol in the community?
   2. What are the facilitators within the health care system to help clients continue Vivitrol in the community?
   3. What are the legal or criminal justice system barriers to clients continuing Vivitrol in the community?
   4. What are the legal or criminal justice system facilitators to clients continuing Vivitrol in the community?
   5. Describe probation and parole officers’ views on Vivitrol and how their views influence Vivitrol use among clients on supervision?

*Possible prompts:*

- - - *What do officers know about Vivitrol?*
    - *What kinds of training do they get on Vivitrol?*

1. For clients who initiate Vivitrol while in prison, we’d like to understand the factors that affect whether or not they continue Vivitrol in the community. Therefore, it is crucial to understand the community treatment process for clients on Vivitrol.
   1. Will you please describe the community treatment process for your last client who initiated Vivitrol® while incarcerated and also continued Vivitrol in the community?

*Possible prompt:*

- - - *What factors helped them access Vivitrol in the community?*
  1. Will you please describe the community treatment process for your last client who initiated Vivitrol while incarcerated but did not continue Vivitrol in the community?

*Possible prompt:*

- - - *What factors hindered their access to Vivitrol in the community?*

1. We’ve talked a lot about individual factors, social networks, and structural factors when it comes to continuing to use Vivitrol in the community. In what ways are these factors similar or different for clients who live in rural areas as compared to urban areas?

BUPRENORPHINE SECTION

1. Next, we’d like to discuss is **buprenorphine**. The KY DOC received funds from the Kentucky Opioid Response Effort (KORE) through the Cabinet for Health & Family Services to offer buprenorphine initiation prior to re-entry for people with OUD incarcerated in Blackburn, Northpoint, and KCIW in 2019.
   1. Describe your experience and/or training with buprenorphine prior to this expansion of the SAMAT protocol.
   2. What are your views on buprenorphine as a treatment for opioid use disorder?
   3. In what ways does buprenorphine help the recovery process for clients with OUD?
   4. In what ways does buprenorphine hinder the recovery process for clients with OUD?
   5. Will you please tell me about any educational materials or training you received on buprenorphine? These could be trainings from DOC on the expanded SAMAT protocol or pharmaceutical companies (e.g., Indivior).
   6. What additional educational materials, trainings, or resources, if any, do you need related to buprenorphine?
2. We’d like to ask you some additional questions about buprenorphine. What types of things do clients say about side effects or other health concerns related to buprenorphine use?
3. You’ve already told us about the factors that influence initiation and continued use of Vivitrol after release. We’d like to compare this to buprenorphine and focus specifically on the characteristics of clients. In what ways are the barriers or facilitators in client characteristics similar or different for buprenorphine?
4. These next questions are related to SAP clients’ social networks. By client’s social network, we are referring to the people your client’s live, work, or socialize with on a regular basis (that is, their family, friends, and acquaintances). We already discussed social network barriers to Vivitrol use.
   1. In comparison to social network barriers to Vivitrol, in what ways are the social network barriers or facilitators similar or different for buprenorphine?
   2. How educated are your client’s family and friends about buprenorphine?

*Possible prompt:*

- - - *What information do people in your client’s social networks have about the costs, side effects, and potential risks and benefits of buprenorphine?*

1. The next set of questions is focused on structural and institutional level factors that may affect the initiation and continued use of buprenorphine for clients transitioning from prison to the community. By structural and institutional factors, we mean things that are larger than the individual or families - like policies and procedures within the KY Department of Corrections, the legal system, healthcare organizations, or insurance companies.
   1. You’ve told us about the barriers and facilitators in the health care and criminal justice system for Vivitrol. In what ways are the barriers and facilitators in these systems similar or different for buprenorphine?

*Possible prompt:*

- - - *You only mentioned (insert either health care system or criminal justice system) factors. In what ways are the (insert either health care system or criminal justice system) similar or different for buprenorphine in comparison to Vivitrol?*
  1. Describe probation and parole officers’ views on buprenorphine and how their views influence buprenorphine use among clients on supervision?

*Possible prompts:*

- - - *Describe what they know about buprenorphine.*
    - *Describe the circumstances in which P&P officers may prohibit or support buprenorphine use.*
    - *What kinds of training do they get on buprenorphine?*
    - *Describe their experiences or concerns with diversion.*

1. Have you had any clients initiate buprenorphine treatment in prison?

***Note: If No, skip to question 19.***

1. For clients who initiate buprenorphine while in prison, we’d like to understand the factors that affect whether or not they continue buprenorphine in the community. Therefore, it is crucial to understand the community treatment process for clients on buprenorphine.
   1. Will you please describe the community treatment process for your last client who initiated buprenorphine while incarcerated and also continued buprenorphine in the community?

*Possible prompt:*

- - - *What factors helped them access buprenorphine in the community?*
  1. Will you please describe the community treatment process for your last client who initiated buprenorphine while incarcerated but did not continue buprenorphine in the community?

*Possible prompt:*

- - - *What factors hindered their access to buprenorphine in the community?*

1. Thanks for talking about the factors influencing buprenorphine use in the community after release. In what ways are these factors similar or different for clients who live in rural areas as compared to urban areas?

METHADONE SECTION

1. Finally, we also want to discuss **methadone**, which is not offered by the DOC.
   1. What are you views on methadone as a treatment for opioid use disorder (OUD)?
   2. In what ways does methadone help the recovery process of clients with OUD?
   3. In what ways does methadone hinder the recovery process of clients with OUD?
   4. What barriers do your clients face when trying to access methadone?

*Possible prompt:*

- - - *What helps clients to overcome these barriers?*

***Interviewer read:*** *Ok, I just have two more questions.*

1. Let’s talk some more about the clients who have initiated either Vivitrol or buprenorphine in prison. How common is it to successfully enroll in Medicaid or some other medical insurance after release in time to access their next medication prescription without your help?

*Possible prompt:*

- - - *For those who don’t, what are the barriers to accessing Medicaid or insurance?*
    - *You only mentioned (insert either Vivitrol or buprenorphine). How common is it to obtain insurance coverage in time to access the next medication prescription for (insert either Vivitrol or buprenorphine)?*

1. What are your suggestions for improving the continuity of medications for the treatment of OUD among individuals re-entering the community in rural areas? What suggestions do you have for improving it in urban areas?

**CONCLUSION**

***Interviewer read****: Now that we have completed the interview questions, we wanted to give you an opportunity to share anything that we might have missed.*

1. Are there any important points that were left out regarding the factors that affect whether or not re-entering clients continue Vivitrol in the community? What about buprenorphine?

**Directions: Thank you for participating in this study. We appreciate your responses.**

**TURN OFF RECODING**

**Handout A:**

**Overview of Medications for the Treatment of Opioid Use Disorder**

**Naltrexone** is an *opioid antagonist*, which means that it works by blocking the activation of opioid receptors. Instead of controlling withdrawal and cravings, it treats opioid use disorder by preventing any opioid drug from producing rewarding effects such as euphoria. It is also used to treat alcohol use disorder.

-FDA approved for the treatment of OUD in 2010

-Trade name is Vivitrol (extended release injection)

**Buprenorphine** is a *partial opioid agonist*, meaning that it binds to those same opioid receptors but activates them less strongly than full agonists do. Like methadone, it can reduce cravings and withdrawal symptoms in a person with an opioid use disorder without producing euphoria.

-FDA approved since 2002

-Trade names: Suboxone (sublingual film or tablet), Zubsolv (sublingual tablet), Sublocade (extended release injection), Probuphine (implant)

**Methadone** is a synthetic *opioid agonist* that eliminates withdrawal symptoms and relieves drug cravings by acting on opioid receptors in the brain – the same receptors that other opioid such as heroin, morphine, and opioid pain medications activate.

-FDA approved since 1972

-Trade names: Methadose (oral concentrate), Dolophine (tablets)
